# Involvement of posterior hypothalamic CaMKII-positive neurons in ADHD-like behaviors in mice

**DOI:** 10.1186/s13041-024-01122-5

**Published:** 2024-08-05

**Authors:** Changwoo Lee, Changsu Woo, Gyeong Ryeong Ma, Kyuhyun Choi, Shin Jung Kang, Ki Soon Shin

**Affiliations:** 1 Department of Biology, Kyung Hee University, Seoul, 02447 Republic of Korea; 2 Department of Integrative Bioscience and Biotechnology, Sejong University, Seoul, 05006 Republic of Korea; 3 Department of Neuroscience, Perelman School of Medicine, University of Pennsylvania, Philadelphia, PA 19104 USA

**Keywords:** Posterior hypothalamus, CaMKII + neurons, Hyperlocomotion, Impulsivity, ADHD

## Abstract

This study explores the behavioral effects of modulating CaMKII-positive (CaMKII+) neurons in the posterior hypothalamus (PH). Utilizing a chemogenetic approach in mice, we discovered that the activation of CaMKII + neurons within the PH is associated with heightened locomotor activity, reduced social interaction, and impulsive behavior unrelated to anxiety or avoidance. These observed behaviors share a significant resemblance with characteristics commonly found in attention deficit and hyperactivity disorder (ADHD). Notably, treatment with clonidine, which is frequently prescribed for ADHD, effectively reduced impulsive behaviors in our mouse model. Our findings uncover the role of the PH that has not been previously explored and suggest a possible involvement of the PH in the manifestation of ADHD-like behaviors.

**Supplementary Information:**

The online version contains supplementary material available at 10.1186/s13041-024-01122-5.

## Introduction

The posterior hypothalamus (PH) resides at the most caudal aspect of the hypothalamus, signifying one of the most ancient and conserved brain regions [1, 2]. The PH plays a crucial role in core survival processes such as regulating body temperature, managing the sleep-wake cycle, controlling aggression, and modulating appetite and sexual desire [3–8]. Given the multifaceted role of the PH, there has been considerable research focus on delineating specific behavioral responses to targeted stimuli and pharmacological agents. For instance, regarding the sleep-wake cycle regulation, research has shown that microinjections of muscimol into the PH not only prolong the duration of sleep but also significantly increase the occurrence of deep slow-wave sleep [9]. Similar increases in sleep time have been observed following optogenetic stimulation of astrocytes in the PH [10]. In research focused on movement, the microinjection of GABA antagonists into the PH elicits defensive behaviors [11, 12]. Likewise, glutamate microinjections into the PH of anesthetized mice initiate locomotor stepping behavior [13], with electrical stimulation producing comparable response [14]. Moreover, numerous lesion studies of the PH have reported that the absence of this region results in decreased aggression [6, 15]. However, the PH contains diverse populations of cell types [1], and such manipulations could simultaneously affect multiple PH neuronal populations. It is plausible that various neuronal types within the PH are associated with unique behavioral responses, emphasizing the importance of investigating behaviors that are dependent on specific neuronal types.

Glutamatergic neurons represent the predominant neuronal population within the PH [1]. To manipulate the activity of this neuronal population within the PH selectively, we utilized adeno-associated viruses (AAV) with a CaMKII promoter to express designer receptors exclusively activated by designer drugs (DREADDs) in CaMKII + glutamatergic neurons in the PH. Following clozapine n-oxide (CNO) administration, the activation or inhibition of PH CaMKII + neurons was efficiently achieved through the employment of DREADD constructs hM3Dq and hM4Di, respectively. Although no changes in emotional behaviors, like anxiety or avoidance, were observed, chemogenetic activation of PH CaMKII + neurons exhibited a significant increase in basal locomotion and a reduction in social interaction. Concurrently, upon the activation of PH CaMKII + neurons, behaviors indicative of risk-taking and impulsivity became apparent as evidenced by the rat exposure test and the cliff avoidance test. These behavioral patterns are suggestive of tendencies commonly associated with attention deficit hyperactivity disorder (ADHD) [16–19]. Intriguingly, these impulsive behaviors were effectively alleviated by clonidine, an a2-adrenergic receptor agonist often used in the treatment of patients with ADHD [20–22]. Our findings indicate that CaMKII + neurons in the PH might play a role in manifestation of ADHD-like behaviors. *To our knowledge*,* present study is the first to demonstrate that the PH activity is associated with ADHD-like behaviors.*

## Results

### Chemogenetic modulation of the activity of CaMKII + neurons in the PH

**Fig. 1:**
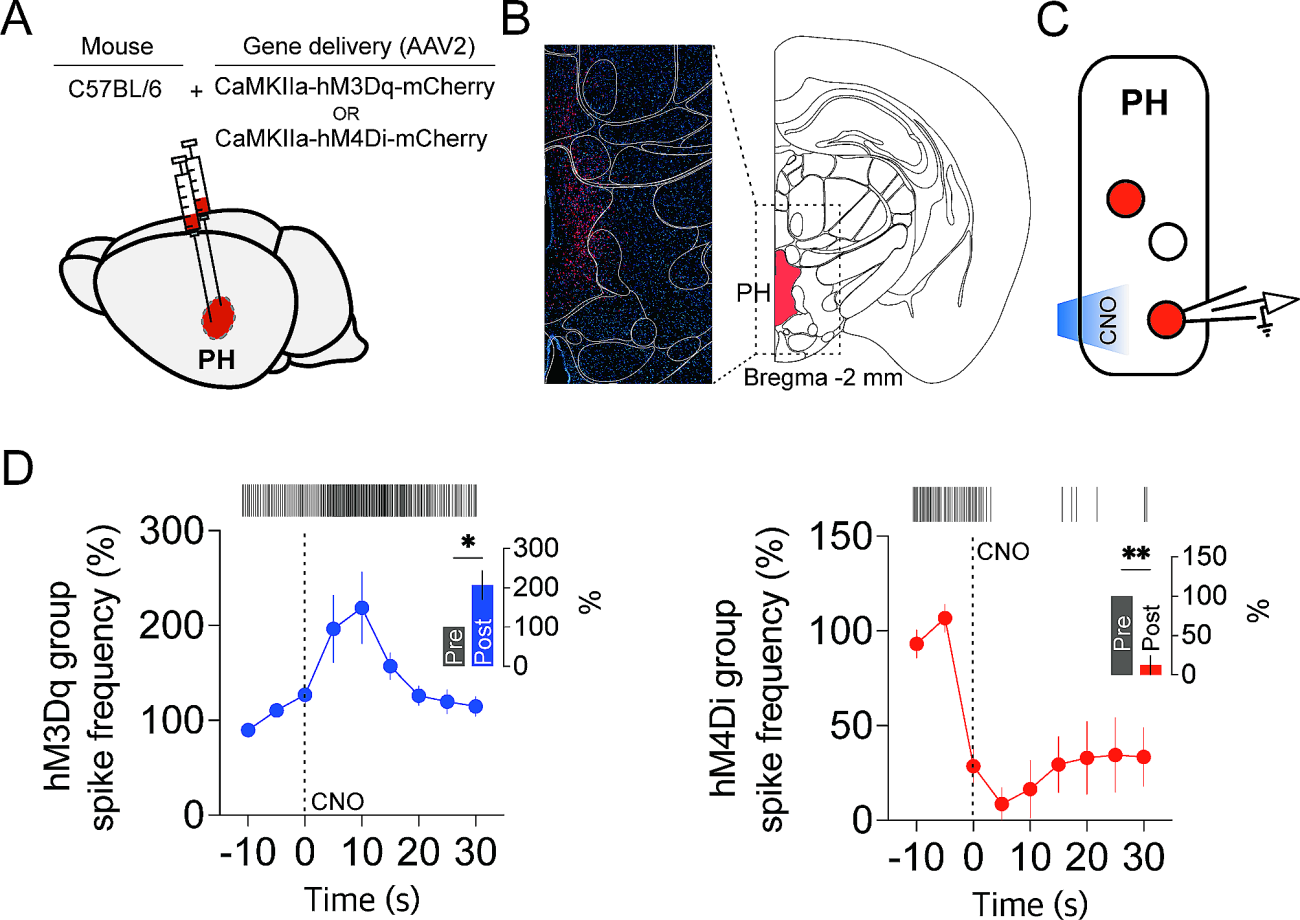
Chemogenetic modulation of the activity of CAMKII + neurons in the PH. (**A**) Schematic for the injection of AAV expressing hM3Dq-mCherry or hM4Di-mCherry into the PH. (**B**) Confocal fluorescence imaging showing the expression of hM3Dq-mCherry in PH CaMKII + neurons (left panel) and the location of brain region on coronally sectioned brain slices at Bregma − 2.0 (right panel). (**C**) Schematic for loose-seal cell-attached patch clamp recordings to assess CNO effects on neuronal activity. (**D**) Action currents (spike) frequency changes following local application of CNO (200 µM) in hM3Dq-expressing mice group (left panel, *n* = 5) and of CNO (500 µM) in hM4Di-expressing mice group (right panel, *n* = 5). CNO was directly applied to the neurons being recorded at 0 s, indicated by the dotted line for 300 ms using a Picospritzer. Spike frequency was normalized by the average value obtained from − 10 s to − 5 s. The raster plots at the top show the temporal locations of action currents. The inset bar graphs compare the frequency of action currents between − 10 s to 0 s with that from 5 s to 15 s (*p = 0.0414, **p = 0.0018, two-tailed paired t-test)

The chemogenetic approach, utilizing DREADDs in conjunction with their specific artificial ligand, CNO, enables precise control of the activity of targeted neurons within well-defined brain areas. Accordingly, we selectively modulated the activity of CaMKII + excitatory neurons in the PH by delivering AAV2-CaMKIIα-hM3Dq-mCherry for activation or AAV2-CaMKIIα-hM4Di-mCherry for inhibition into the PH via stereotaxic surgery (Fig. 1A). Four weeks after the surgery, hM3Dq- or hM4Di-expressing CaMKII + neurons in the PH were visibly marked by mCherry fluorescent proteins (Fig. 1B). To verify the effect of CNO on the activity of neurons expressing hM3Dq or hM4Di, we performed loose-seal cell-attached voltage-clamp recordings on mCherry-labeled neurons and measured spontaneous action currents (Fig. 1C). We directly applied CNO just adjacent to the neurons being recorded for 300 ms using a Picospritzer. Neurons expressing hM3Dq showed an increase in action current frequency following CNO application, peaking after 10–15 s and returning to baseline after 20 s (Fig. 1D, left panel). In contrast, neurons expressing hM4Di demonstrated a decrease in action current frequency following CNO application (Fig. 1D, right panel). These results indicate that CaMKII + neurons in the PH could be activated and inhibited chemogenetically through the activation of hM3Dq and hM4Di, respectively.

### Activation of CaMKII + neurons in the PH produced hyperactivity in locomotion

**Fig. 2:**
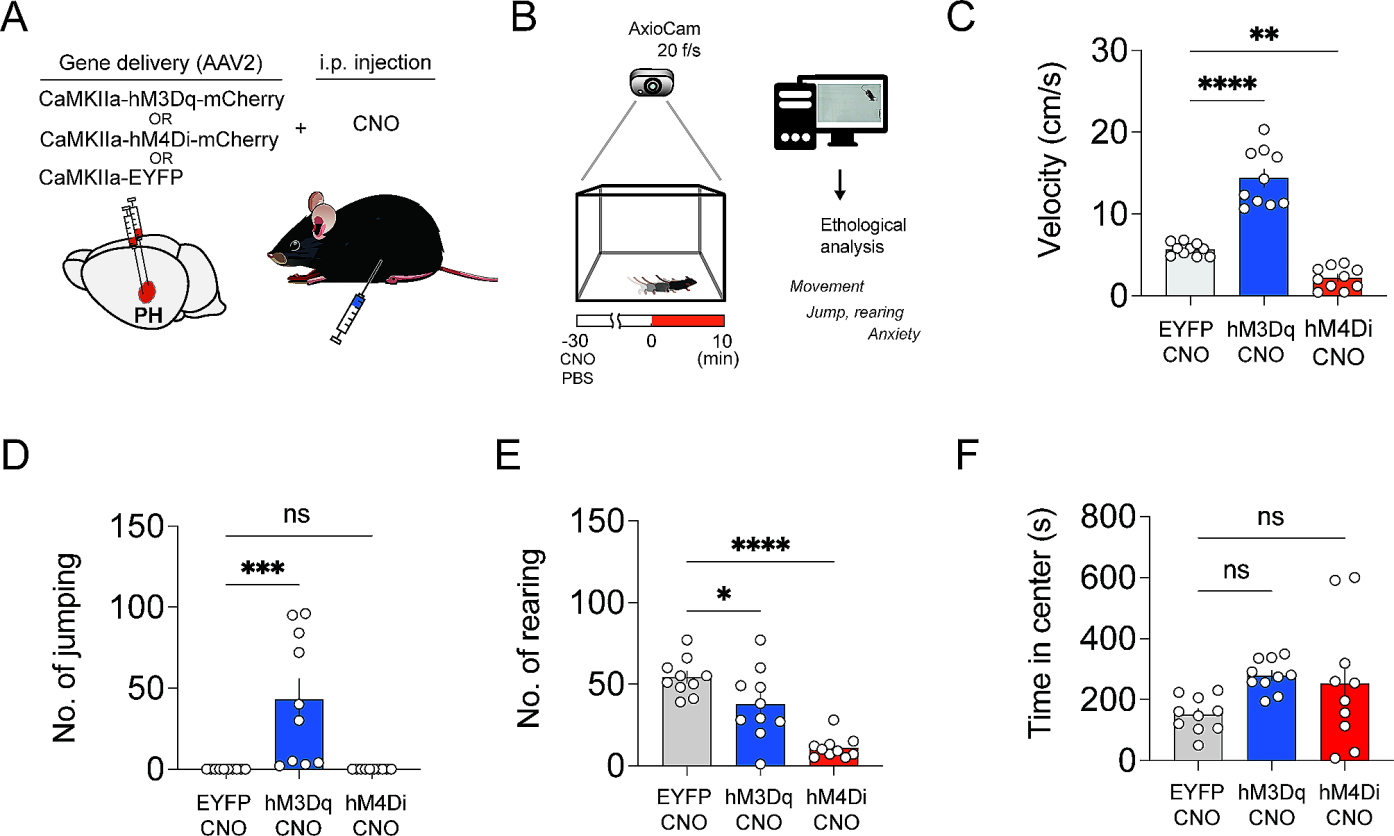
Activation of CaMKII + neurons in the PH produced hyperactivity in locomotion. (**A**) Schematic showing the chemogenetic control of activity of PH CaMKII + neurons. The hM3Dq-CNO group (*n* = 10) received an i.p. injection of CNO (1.5 mg/kg) in mice expressing hM3Dq-mCherry in the PH. The hM4Di-CNO group (*n* = 10) received an i.p. injection of CNO (5 mg/kg) in mice expressing hM4Di-mCherry in the PH. The control group (EYFP-CNO, *n* = 10)) received an i.p. injection of CNO (5 mg/kg) in mice expressing EYFP in the PH. (**B**) Schematic for the open field test. CNO was i.p. injected 30 min before the open field recording. (**C**) Differences in velocity (****p < 0.0001, **p = 0.0028, one-way ANOVA with Bonferroni’s post hoc test). (**D**) Differences in the number of jumping behavior (***p = 0.0006, one-way ANOVA with Bonferroni’s post hoc test). (**E**) Differences in rearing frequency (*p = 0.036, ****p < 0.0001, one-way ANOVA with Bonferroni’s post hoc test). (**F**) No differences in center duration. All values are presented as mean ± SEM

To assess how modulation of activity of CaMKII + neuron in the PH affects mouse behavior, *AAV2-CaMKIIα-hM3Dq-mCherry*,* AAV2-CaMKIIα-hM4Di-mCherry or AAV2-CaMKIIα-EYFP* delivered mice were administered CNO intraperitoneal (i.p.) injection. Mice expressing hM3Dq received 1.5 mg/kg CNO (*hM3Dq-CNO*), while those expressing hM4Di received 5 mg/kg CNO (*hM4Di-CNO group). For the control*,* mice expressing EYFP received 5 mg/kg CNO (EYFP-CNO group).* Schematics are shown in Fig. 2A. Thirty minutes after the administration, the mice were allowed to freely explore an open field area for 10 min (Fig. 2B). The hM3Dq-CNO group exhibited significantly increased movement, with their velocity more than doubling that of the control group. Conversely, the hM4Di-CNO group showed reduced movement, with extreme instances of some mouse remaining stationary for the duration of the entire recording time (Fig. 2C). One-way ANOVA revealed a significant effect of group *(F*_*2,27*_*= 81.69*,*P < 0.0001)*. Additionally, the hM3Dq-CNO mouse group exhibited repeated strong jumps towards the ceiling in the open field (Fig. 2D; *F*_*2,27*_*= 11.54*,*P < 0.001*, one-way ANOVA). However, neither *EYFP-CNO* nor the hM4Di-CNO groups displayed such jumping behavior (Fig. 2D). To determine if this hyperactivity was due to heightened anxiety, we compared the frequency of rearing and the center duration. *The number of rearing events rather decreased in both hM3Dq-CNO and hM4Di-CNO groups compared to EYFP-CNO group* (Fig. 2E; *F*_*2,27*_*= 22.0*,*P < 0.0001*, one-way ANOVA). In addition, no significant differences in center duration were found among the groups (Fig. 2F; *F*_*2,27*_*= 2.81*,*P = 0.078*, one-way ANOVA). *Therefore*,* the hyperactivity resulting from the chemogenetic activation of PH CaMKII + neurons may not be due to heightened anxiety.*

Although there were no differences in anxiety levels among the groups, the significantly increased movement and repetitive jumping toward the ceiling in the hM3Dq-CNO group led us to speculate about potential aversive behavior. To investigate this, we conducted a conditioned place avoidance (CPA) test. Contrary to our expectation, there were no significant changes in chamber preference after conditioning (see Supplementary Fig. S1). Thus, we concluded that the hyperactivity observed in the hM3Dq-CNO group was not associated with aversive emotional value. Furthermore, activity of PH CaMKII + neurons did not affect working memory, evidenced by Y-maze test (see Supplementary Fig. S2).

### Activation of CaMKII + neurons in the PH reduced sociality

**Fig. 3:**
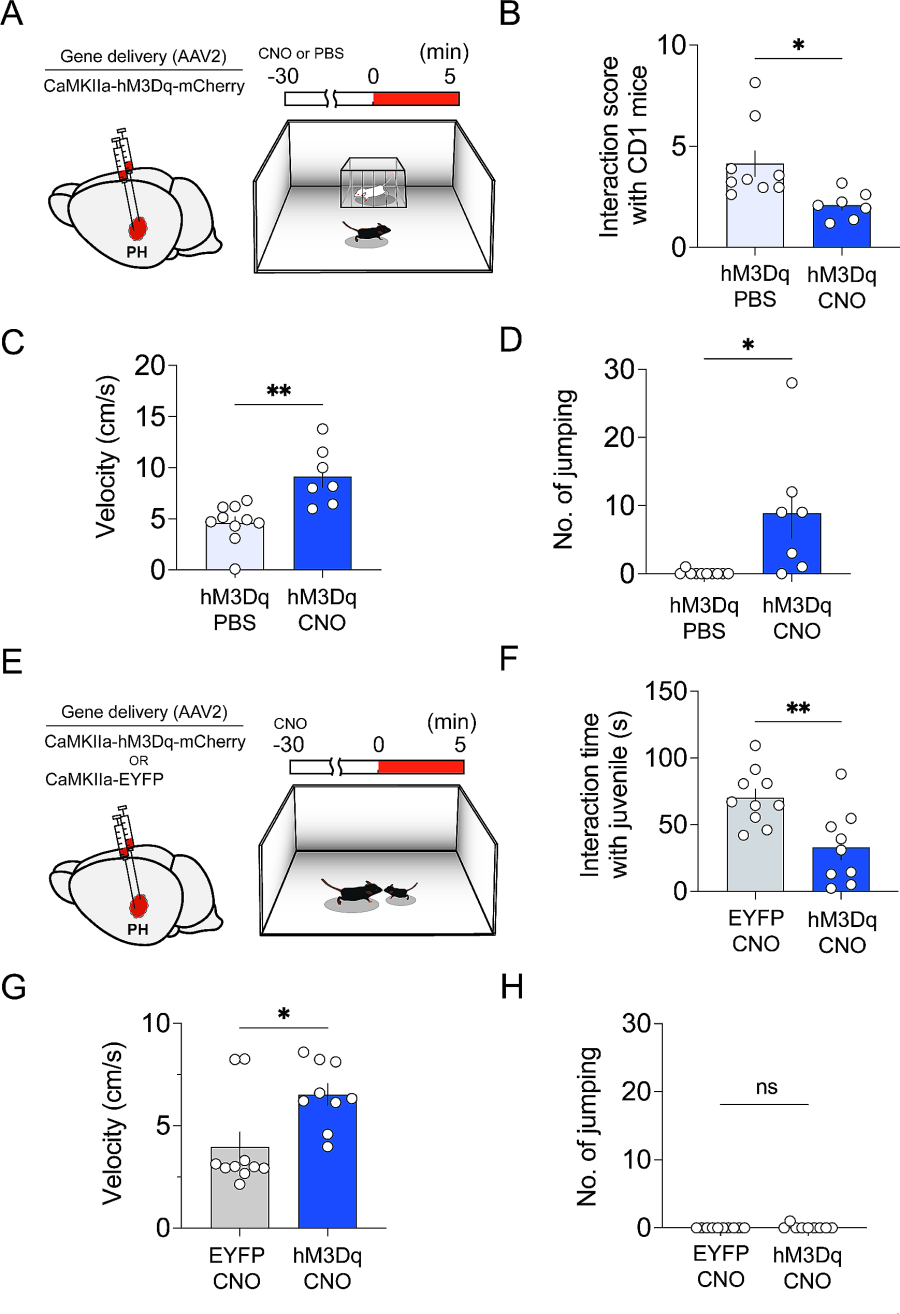
Activation of CaMKII + neurons in the PH reduced sociality. (**A**) Schematics for AAV injection (left panel) and the social interaction test with CD1 mice (right panel). The hM3Dq-PBS group received an i.p. injection of PBS (*n* = 9). The hM3Dq-CNO group received an i.p. injection of CNO (1.5 mg/kg, *n* = 7). (**B**) Social interaction scores of hM3Dq-PBS and hM3Dq-CNO groups with CD1 (*p < 0.05, two-tailed unpaired t-test). (**C**) Difference in velocity during the social interaction test with CD1 (**p = 0.0012, *two-tailed unpaired t-test).* (**D**) Differences in the number of jumping behaviors in the context of social interaction with CD1 (*p = 0.010,* two-tailed unpaired t-test).* (**E**) Schematics for AAV injection (left panel) and the social interaction test with C57BL/6 juvenile mice (right panel). The EYFP-CNO group (*n* = 10) and hM3Dq-CNO group (*n* = 9) received an i.p. injection of CNO (1.5 mg/kg). (**F**) Difference in the interaction time of hM3Dq-CNO and EYFP-CNO groups in the social interaction test with C57BL/6 juvenile mice (**p = 0.004, two-tailed unpaired t-test). (**G**) Difference in velocity during the social interaction test with C57BL/6 juvenile mice (*p = 0.012, *two-tailed unpaired t-test). (***H**) *Difference in the number of jumping behaviors in the context of social interaction with C57BL/6 juvenile mice (p = 0.31*,* two-tailed unpaired t-test)*

Given the hM4Di-CNO group showed a minor reduction in movement and absence of other notable behavioral differences, subsequent research focused on the hM3Dq group. Now, the effect of activating CaMKII + neurons in the PH were explored by evaluating social behavior, specifically by measuring the interactions with unfamiliar CD1 mice (Fig. 3A). *For the control*,* mice expressing hM3Dq received PBS (hM3Dq-PBS). With the CD1 mouse*,* the hM3Dq-CNO group showed a lower social interaction score compared to the hM3Dq-PBS group* (Fig. 3B). The social interactions with unfamiliar juvenile C57BL/6 mice were also evaluated (Fig. 3E). *As the control (EYFP-CNO) for this experiment*,* CNO was i.p. injected into the mice expressing EYFP in CaMKII + neurons in the PH.* Just as in the interaction with CD1 mouse, the hM3Dq-CNO group less interacted with juvenile C57BL/6 mouse compared to the EYFP-CNO group (Fig. 3D). These results demonstrated that the activation of CaMKII + neurons in the PH reduces sociality.

*It has been reported that adolescent ADHD patients exhibit reduced social motivation and increased social anxiety* [23]. *To assert that the decreased social behavior observed in hM3Dq-CNO group constitutes ADHD-like behavior*,* we analyzed locomotor activity and jumping behavior during the social interaction tests. The hM3Dq-CNO group exhibited significantly increased movement even in the social contexts (*Fig. 3C *and G). Moreover*,* the hM3Dq-CNO mouse group exhibited repeated jumping during the social interaction with CD1 (*Fig. 3D*) but not with juvenile C57BL/6 (*Fig. 3H*). Therefore*,* the decreased sociality in hM3Dq-CNO group presumed to be indicative of ADHD-like behavior.*

### Activation of CaMKII + neurons in the PH produces impulsive behavior

**Fig. 4:**
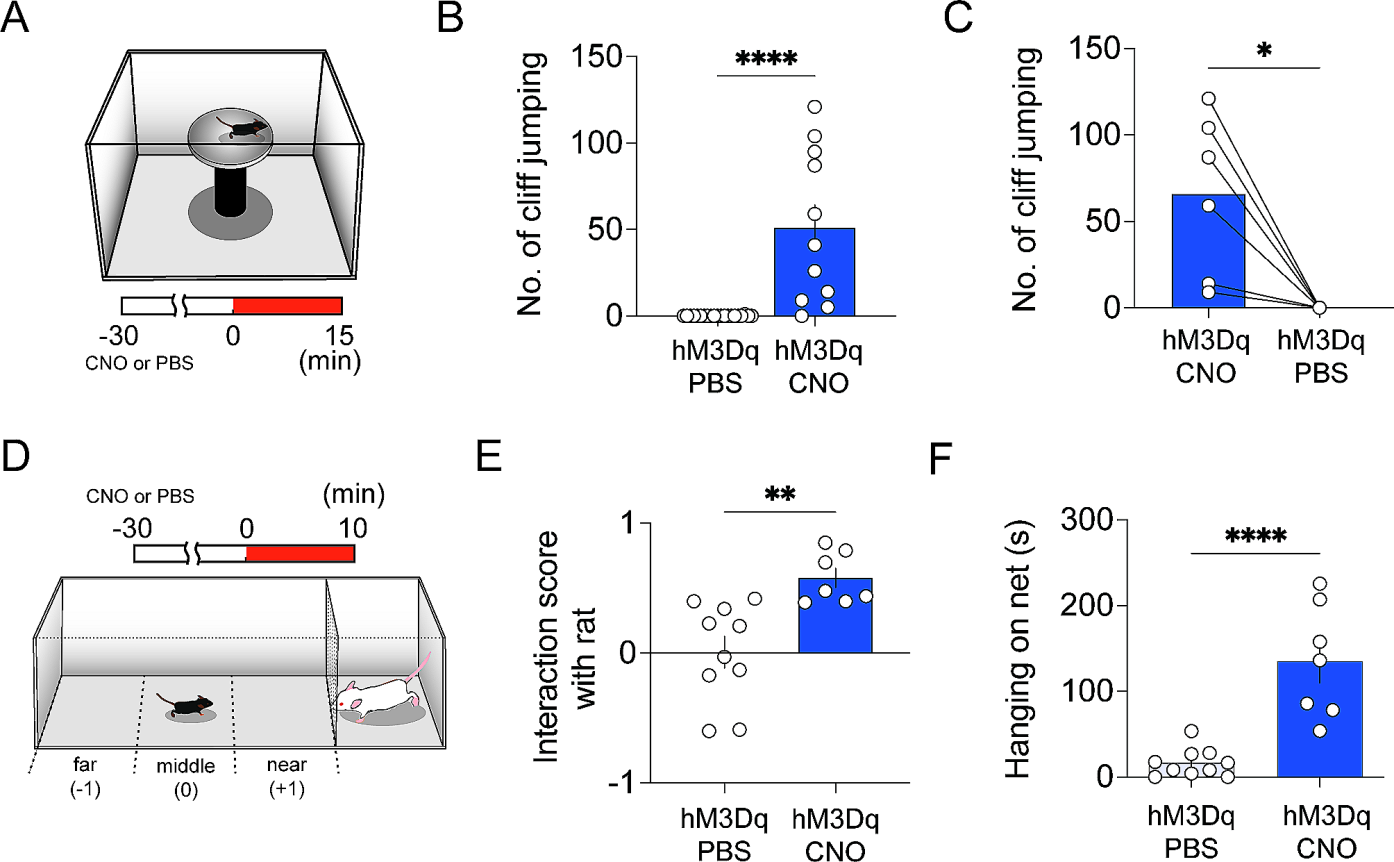
Activation of CaMKII + neurons in the PH produces impulsive behavior. (**A**) Schematic diagram of the cliff avoidance test. (**B**) The number of jumps down from the cliff was recorded for the hM3Dq-CNO group (*n* = 11) and the hM3Dq-PBS (*n* = 21), revealing a statistically significant difference (****p < 0.0001, two-tailed unpaired t-test). (**C**) After administrating i.p. injection of CNO to mice expressing hM3Dq (hM3Dq-CNO) and performing cliff avoidance test, the same mice were injected with PBS the next day (hM3Dq-PBS), followed by a subsequent cliff avoidance test. There was a significant difference in the number jumping-down behaviors between the groups (*n* = 6, *p < 0.05, two-tailed paired t-test). (**D**) Schematic diagram of the rat exposure test. (**E**) The interaction scores of hM3Dq-PBS (*n* = 10) and hM3Dq-CNO (*n* = 7) groups in the rat exposure test (**p < 0.01, two-tailed unpaired t-test). (**F**) Time hM3Dq-PBS (*n* = 10) and hM3Dq-CNO (*n* = 7) mice hung on the net in front of the rat (****p < 0.0001, two-tailed unpaired t-test)

Considering the pronounced hyperactivity, repetitive jumping, and reduced social interactions observed in hM3Dq-CNO group, we speculated that these behaviors might indicate ADHD. To further investigate this possibility, we conducted tests on impulsive behavior, which is characterized by atypical unpredictable, or risky actions that deviate from instinct and are often indicative of ADHD-like behavior. For this purpose, we carried out a cliff avoidance test by placing mice on an elevated platform to see if they would jump off (Fig. 4A) and a rat exposure test to observe their reactions when placed in the same space with a large rat as a predator (Fig. 4D).

In the cliff avoidance test, *the control group mice (hM3Dq-PBS)* did not jump off the platform, whereas the hM3Dq-CNO mice repeatedly leaped from the cliff (Fig. 4B). Since we observed the hM3Dq-expressing mice jumping off a cliff after injecting CNO, the following day, we injected PBS instead of CNO in the same mice and monitored their jumping behavior again. As shown in Fig. 4C, the mice that had jumped the previous day showed no such behavior. These findings suggest that the activation of PH CaMKII + neurons is closely related to the impulsive behavior of jumping off cliffs.

During the rat exposure test, the hM3Dq-PBS control group maintained a distance and remained vigilant around the rat, while the hM3Dq-CNO mice showed little fear, staying in the near zone, and even climbed the netting in front of the rat (Fig. 4E, F). These results reveal that the activation of CaMKII + neurons in the PH leads to impulsive behavior.

### Clonidine reversed the impulsive behavior but did not affect hyperlocomotion

**Fig. 5:**
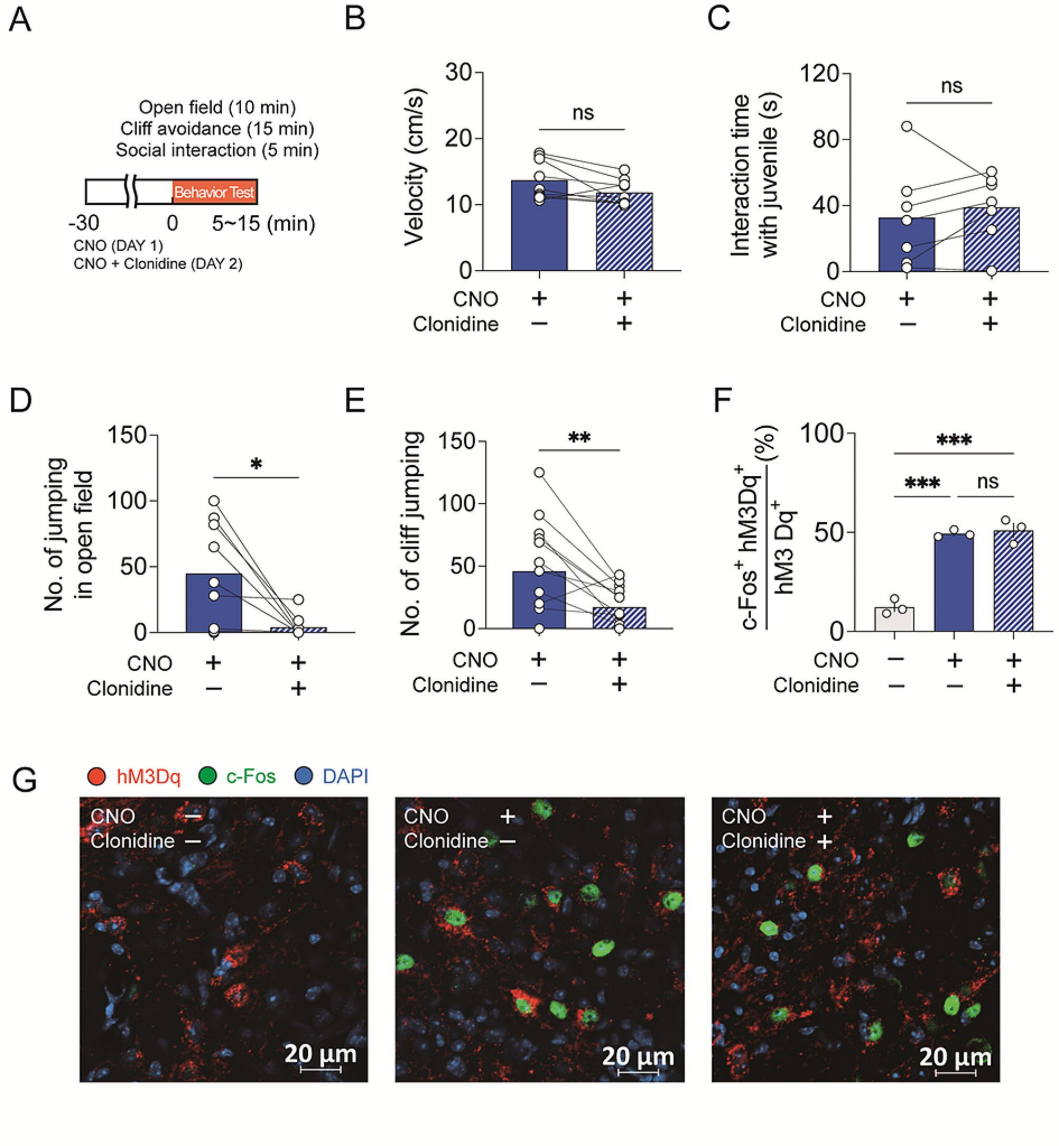
Clonidine reversed the impulsive behavior but not hyper-locomotion produced by the activation of PH CaMKII + neurons. (**A**) Schematic diagram for behavioral tests. The mouse group injected with AAV-CaMKIIa-hM3Dq-mCherry into the PH was utilized. After administrating i.p. injection of CNO (1.5 mg/kg) to the mice and conducting behavioral tests, the same mice were given a combined injection of CNO and clonidine (1 mg/kg) the next day, followed by behavioral tests. (**B**) Moving velocity of the hM3Dq-CNO mice with or without clonidine injection (*n* = 9, *p* = 0.057, two-tailed paired t-test). (**C**) No change in interaction time with juvenile C57BL/6 mice of hM3Dq-CNO mice by clonidine (*n* = 7, *p* = 0.4364, two-tailed paired t-test). (**D**) Decrease of the number of jumping behavior of the hM3Dq-CNO mice in the open field induced by clonidine (*n* = 9, *p = 0.016). (**E**) Decrease in the number of cliff-jumping events of hM3Dq-CNO mice induced by clonidine (*n* = 13, **p = 0.008, two-tailed paired t-test). (**F**) *No change by clonidine in the percentage of neurons expressing c-Fos among hM3Dq –expressing CaMKII + neurons in the PH. The hM3Dq-expressing mouse group was administrating i.p. injection of CNO or a combined injection of CNO and clonidine. After one hour*, brains were processed for immunohistochemistry. The percentage of c-Fos-positive neurons among approximately 200 hM3Dq-mCherry-positive neurons was estimated for each animal. Average percentage values were then calculated from three animals per experimental group (***p < 0.001, *one-way ANOVA with Bonferroni’s post hoc test). (***G***) Representative images showing c-Fos (green) expression in hM3Dq-mCherry (red) expressing neurons in the PH. Nuclei were stained with DAPI (blue)*

Clonidine is a medication used to manage overactivity and impulsivity in ADHD patients. Based on this, we tested whether clonidine reverses ADHD-like behaviors found in our hM3Dq-CNO group. As shown in Fig. 5A, clonidine (1 mg/kg) was co-administered with CNO in hM3Dq-expressing mice, and we repeated behavioral tests assessing hyperactivity, impulsivity, and sociality. In mice expressing hM3Dq, there was no difference in velocity whether CNO was administered alone or together with clonidine (Fig. 5B). There was also no improvement observed in the social interaction with juvenile C57BL/6 mice (Fig. 5C). However, clonidine treatment significantly suppressed repetitive jumping behavior in the open field (Fig. 5D) and effectively reduced jumping from the cliff (Fig. 5E). These results suggest that clonidine can effectively suppress impulsive behaviors.

*We investigated whether clonidine suppresses impulsive behaviors by reducing the activity of PH CaMKII + neurons directly. To visualize neuronal activity in the PH*,* we measured c-Fos expression in hM3Dq-expressing CaMKII + neurons within the PH. One hour after i.p. injection of CNO or CNO combined with clonidine*,* the mice were sacrificed*,* and c-Fos immunostaining was performed. As shown in* Fig. 5F *and G*,* activation of CaMKII + neurons by CNO administration induced c-Fos expression (F*_*2,6*_*= 75.74*,*P < 0.0001*,* one-way ANOVA). Meanwhile*,* clonidine did not suppress this hM3Dq activity-induced c-Fos expression. Our findings suggest that ADHD-like behaviors observed following PH activation are likely not solely attributable to PH activation in isolation but rather involve the modulation of other brain regions by the PH.*

## Discussion

ADHD is a common mental disorder affecting 5% of children globally [17], presenting significant challenges in daily life for those affected and their family members. Despite its prevalence, the underlying causes of ADHD remain largely unexplained [17, 20]. Diagnoses of ADHD do not rely on specific genetic markers but rather on the frequency of certain behavioral patterns (e.g., hyperactivity, attention deficit, impulsive behavior, and reduced sociality) [17, 18, 23], which are similarly observed in ADHD mouse models. While various mouse models exist for ADHD research, they do not capture all ADHD-related behaviors. Typically, these models exhibit hyperactivity, and some additionally show diminished social interaction, impulsive behavior, or attention deficits [24, 25].

Our study observed diverse behavioral changes in mice through the manipulation of CaMKII + neuron activity in the PH. Notably, increased activity of CaMKII + neurons in the PH dramatically increased mouse locomotion and exhibited behaviors such as leaping from cliffs and approaching predators without fear. These actions can be described as risk-taking or impulsivity and go against evolutionary instincts [26, 27]. PH CaMKII + neuronal activation also demonstrated lower sociality, closely mirroring the behavioral patterns observed in ADHD [17, 24].

Clonidine, an alpha-2 adrenergic receptor agonist, has been known for effectively reducing impulsive behavior in ADHD patients [20–22]. Therefore, it is noteworthy of the effect of clonidine in our study. We found that clonidine selectively diminished the impulsive behavior in the hM3Dq-CNO group, significantly lowering the frequency of jumping from cliffs or repetitive leaping in the open field. On the other hand, we found that clonidine did not affect the hyperactivity and lack of sociality in the hM3Dq-CNO group. Considering clonidine’s selective suppression of behavioral patterns, it’s plausible that the ADHD-like behaviors in hM3Dq-CNO mice might involve multiple brain regions. *Supporting this speculation*,* clonidine did not attenuate PH neuronal activity in hM3Dq-CNO group*,* as indicated by c-Fos expression. Therefore*,* ADHD-like behaviors observed in the hM3Dq-CNO group are likely mediated by the modulation of other brain regions in conjunction with the PH.*

We speculate on how the activity changes in the PH could manifest such behaviors through specific sub-brain regions. Utilizing connectivity data from the Allen Brain Atlas [28], we identified strong projections from the PH to regions such as the lateral septum (LS), nucleus reuniens, zona incerta, and dorsal raphe (DR). Among these, the LS, known for its activation of dopaminergic neurons causing hyperlocomotion [29], might be the underlying region for hyperlocomotion in the hM3Dq-CNO model, suggesting that excitatory neuron activity in the PH increases DAT + neuron activity in the LS, inducing hyperlocomotion. Meanwhile, impulsive behavior might be mediated through the DR, a region implicated in ADHD’s impulsive behaviors [30]. Inhibition of 5-HT neurons within this area has been reported to produce impulsive behaviors [31], and treatments for ADHD patients’ impulsive behaviors often involve increasing serotonin levels through selective serotonin reuptake inhibitors [20]. The DR also contains a significant proportion of GABAergic neurons [32]; if impulsive behavior in hM3Dq-CNO mice is mediated through this region, it suggests that the activity of excitatory neurons in the PH could induce impulsive behavior by feed-forward inhibition of serotonergic neurons via GABAergic neurons. Further research is necessary to closely examine the relationship between these candidate regions and ADHD-like behaviors to elucidate the gating mechanisms behind these behaviors. We anticipate that our research will contribute to understanding ADHD’s mechanisms and aid in research towards alleviating its symptoms.

## Materials and methods

### Animals

Experiments received ethical clearance from the Kyung Hee University Animal Care and Use Committee (Approval ID: KHUASP-22-453). The study utilized male C57BL/6 mice, CD1 mice and SD rats. Animals were group-housed under a 12-hour light/dark cycle at 24 °C and 30–60% humidity, with *ad libitum* access to food and water.,

### AAV injection

Five-week-old C57BL/6 mice were used for stereotaxic AAV injection. AAVs including AAV2-CaMKIIa-hM3Dq-mCherry or AAV2-CaMKIIa-hM4Di-mCherry were purchased from ADDGENE. Additionally, AAV5-CaMKIIa-eYFP was obtained from the UNC Vector Core. A volume of 0.6 µL per virus was injected into the target regions using Picospritzer III (Parker). Mice were respiratory anesthetized with isoflurane and bupivacaine (5 mg/kg) was treated for pain relief. Surgical procedures were conducted on a stereotaxic frame (Stoelting). The injections targeted the PH (AP -2.0 mm, ML ± 0.25 mm, DV -4.7 mm). Experimental protocols commenced 4–5 weeks post-surgery.

### Behavioral tests

#### Intraperitoneal (i.p.) injection

All mice used in the experiments were i.p. injected with either CNO or clonidine 30 min prior to the behavioral tests. CNO was administered at a concentration of 1.5 mg/kg for mice expressing hM3Dq, and at 5 mg/kg for those expressing hM4Di or EYFP. Clonidine was injected at a concentration of 1 mg/kg. All drugs were prepared in a PBS-based solution.

#### Open field test

Locomotor activity was assessed in a 40 × 40 × 30 cm square enclosure. The mice’s positions over time were analyzed using EthoVision 3.1 (Noldus), yielding coordinates, velocity, and distance traveled.

#### Social interaction test with CD1 mice

The transparent perforated plastic cage was placed against the center of one wall in the open field. Initially, a mouse was placed in the arena to measure its exploratory behavior towards the empty cage for a duration of 150 s. After this initial period, a CD1 mouse was introduced into the perforated cage, and the exploration time of the C57BL/6 mouse near the cage now containing the CD1 mouse was observed for another 150 s. The social interaction score was calculated as the ratio of the exploration time adjacent to the cage with the CD1 mouse to that during the empty cage phase.

#### Social interaction test with juvenile mice

The open field arena was used to evaluate the social behavior of experimental group mice with juvenile C57BL/6 mice. The juvenile mice employed in this study were 4 weeks old. Social interactions were quantified by measuring the cumulative duration of direct nasal contact and chasing behaviors exhibited by the experimental mice towards the juveniles. The total interaction time was the sum of these two behaviors, providing a comprehensive assessment of the active engagement between the mice during the testing period.

#### Cliff avoidance test

The circular platform measuring 11 cm in diameter was set at an elevation of 15 cm above the ground. A mouse was placed atop this platform. If the mouse leaped off, it was manually retrieved and returned to the platform by the experimenter. This process was repeated throughout the 15-min testing period.

#### Rat exposure test

The rectangular chamber, divided by wire mesh into two areas, housed a rat on one side as a potential predator. In the opposite section, a mouse was introduced to assess its interaction with the predator presence. The mouse-occupied section was divided into three virtual zones to measure proximity to the predator: -1 point for the farthest zone, 0 points for the neutral middle, and + 1 point for the closest zone. An interaction score was calculated by multiplying the time the mouse spent in each zone by the zone’s assigned value, quantifying the mouse’s avoidance or approach behavior.

#### Conditioned place aversion (CPA)

The CPA apparatus comprised two interconnected sections, each with distinct patterns (stripes and checkered). A mouse explored the chambers for 15 min on the first day to establish baseline preferences (pre-preference test). Conditioning occurred over the next four days. Before the pairing of CNO injection with the preferred chamber, PBS were administered in the non-preferred chamber. Each session lasted for 10 min in each chamber. Preference reassessment was conducted on the sixth day (post-preference test).

#### Y-maze test

A mouse was placed within a maze featuring three plastic arms set at 120° angles to evaluate spatial memory. Over the course of 10 min, the mouse was observed as it moved freely through the arms. Mice characteristically prefer to explore a less recently visited arm, indicative of working memory function. The percentage of correct arm entries was determined using the formula: [(number of alternations) / (total arm entries − 2)] × 100, where an alternation represents a consecutive entry into all three arms without repetition.

### Acute slice preparation

Mice were deeply anesthetized with isoflurane, followed by myocardial perfusion using 4℃ aCSF with the following composition (in mM): 124 NaCl, 2.5 KCl, 1.2 NaH_2_PO_4_, 24 NaHCO_3_, 5 HEPES, 13 Glucose, 2 MgSO_4_, 2 CaCl_2_. Subsequently, the brain was promptly removed and coronally sectioned on a vibratome (VT1000s, Leica) into 250 μm slices submerged in 4℃ aCSF. These slices were then expeditiously transferred to a 32℃ recovery solution with the following composition (in mM): 92 NMDG, 2.5 KCl, 1.2 NaH_2_PO_4_, 30 NaHCO_3_, 20 HEPES, 25 Glucose, 5 Sodium ascorbate, 2 Thiourea, 3 Sodium pyruvate, 10 MgSO_4_, 0.5 CaCl_2_. After 12 min of recovery, the slices were transferred to room temperature aCSF.

### Electrophysiology

Electrophysiological recordings were conducted using an EPC10 amplifier (HEKA), with signals sampled at 10 kHz. Patch-clamp pipettes were fabricated from borosilicate glass (Warner Instruments) and exhibited a tip resistance of approximately 5 MΩ when filled with internal solution. Recovered slices were transferred to the recording chamber, which was submerged in 30 °C aCSF flowing at a rate of 1.6 mL/min. Cell observation was facilitated using a 40x magnification video microscope (BX51WI, Olympus). Action currents recordings were performed using the loose-seal cell-attached patch-clamp configuration with pipettes filled with aCSF. Data analysis was performed using Patchmaster (HEKA) and Igor 6.0 (Wavemetrics Inc.).

### Confocal imaging

Mouse brains, post-fixed with 4% paraformaldehyde in PBS, were sectioned coronally at 30 μm thickness using a Cryotome FSE (Thermo Scientific). *For c-Fos immunostaining*,* brain slices were permeabilized in 0.5% Triton X-100 for 20 min and blocked in 5% normal serum in PBS for 1 h 30 min in free floating condition. Anti-c-Fos antibody (1:500*,* Santa Cruz) were incubated for overnight in 3% normal donkey serum and 0.3% triton X-100 in PBS at 4 °C. For visualization of primary antibodies*,* slices were incubated with Alexa 488-anti-rabbit secondary antibodies (1:500*,* Molecular probes) for 40 min. To visualize endogenous fluorescence from AAV-expressed proteins (mCherry)*,* c-Fos (Alexa 488) and nuclear staining (DAPI*,* 4’*,*6-diamidino-2-phenylindole*,* Sigma-Aldrich)*,* slices were counterstained with DAPI and imaged with a confocal laser scanning microscope (LSM 800*,* Carl Zeiss*).

### Statistical analysis

The data in the figures are presented as means ± standard error of the mean (SEM). Statistical significance was determined by calculating *p*-values using Student’s t-tests or analysis of variance (ANOVA), as appropriate. Statistical analyses and creation of graphs were performed using GraphPad Prism 9.5.0 (GraphPad Software Inc.).

### Electronic supplementary material

Below is the link to the electronic supplementary material.

Supplementary Material 1

## Data Availability

All data generated in this study are included in this article.

## Abbreviations

PH: Posterior hypothalamus
ADHD: Attention deficit and hyperactivity disorder
AAV: Adeno-associated viruses
DREADDs: Designed receptors exclusively activated by designer drugs
CNO: Clozapine n-oxide
CPA: Conditioned place avoidance
LS: Lateral septum
DR: Dorsal raphe
