# Supplementary material for: Involvement of posterior hypothalamic CaMKII-positive neurons in ADHD-like behaviors in mice
Source: Mol Brain. 2024 Aug 5;17:51. doi: 10.1186/s13041-024-01122-5 (PMC11302079; doi:10.1186/s13041-024-01122-5)
Supplement: Supplementary file 1 — Supplementary Material 1 [file 13041_2024_1122_MOESM1_ESM.docx]

**Supplementary**

**
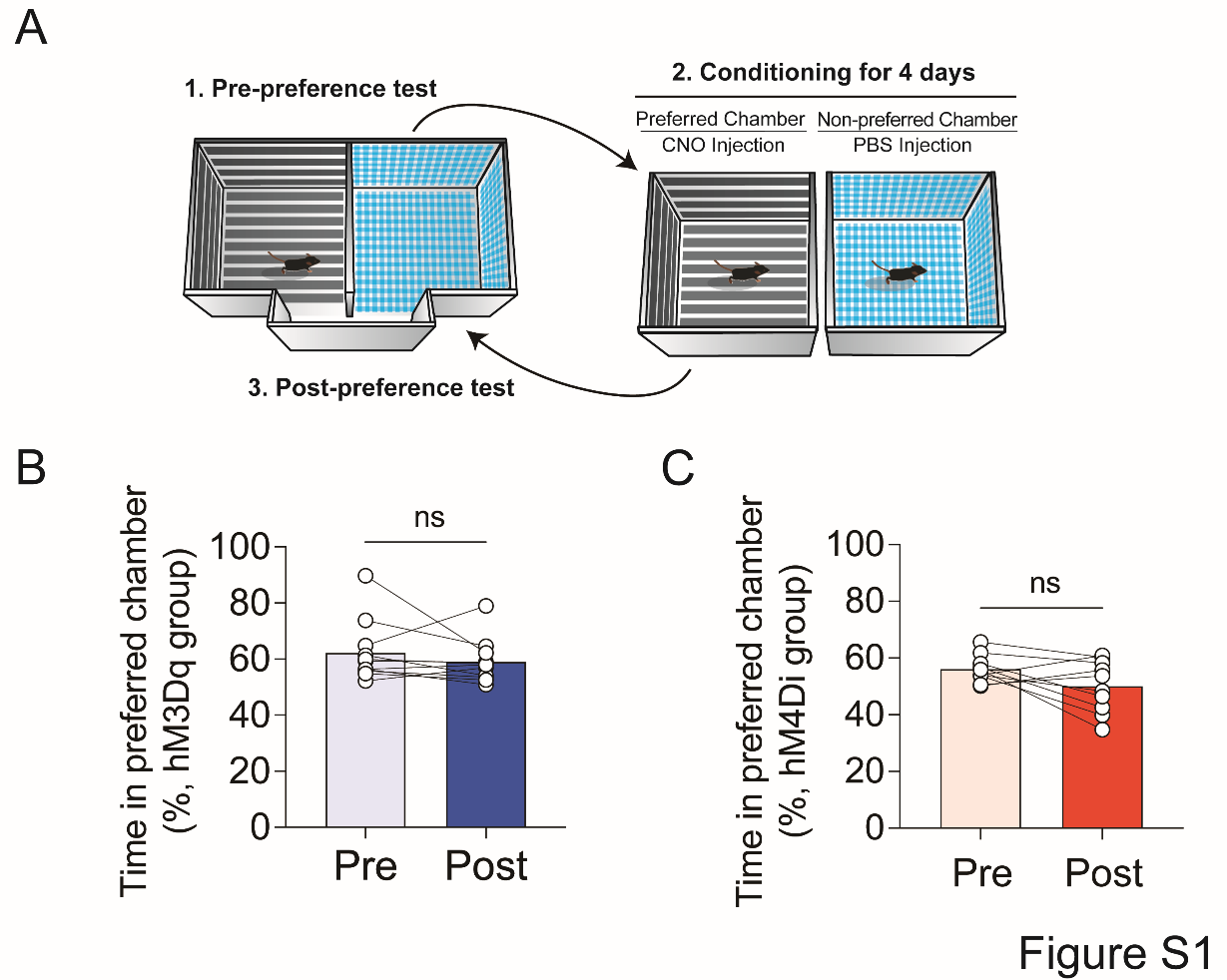
**

Figure S1. Activity of CaMKII+ neurons in the PH was not involved in aversion. (A) Viral injection strategy (left panel) and schematic diagram of the conditioned place avoidance test (right panel). During the 4-day conditioning period, mice in the hM3Dq and hM4Di groups were placed daily in the non-preferred chamber (identified in the pre-preference test) for 10 min after receiving a PBS injection, followed by 10 min in their preferred chamber after a CNO injection. PBS or CNO was i.p. injected 30 min prior to the conditioning. (B) No shift in place preference was observed following the place conditioning in the hM3Dq group (p = 0.396, two-tailed paired t-test, n = 11). (C) No shift in place preference was observed following the place conditioning in the hM4Di group (p = 0.378, two-tailed paired t-test, n = 10).


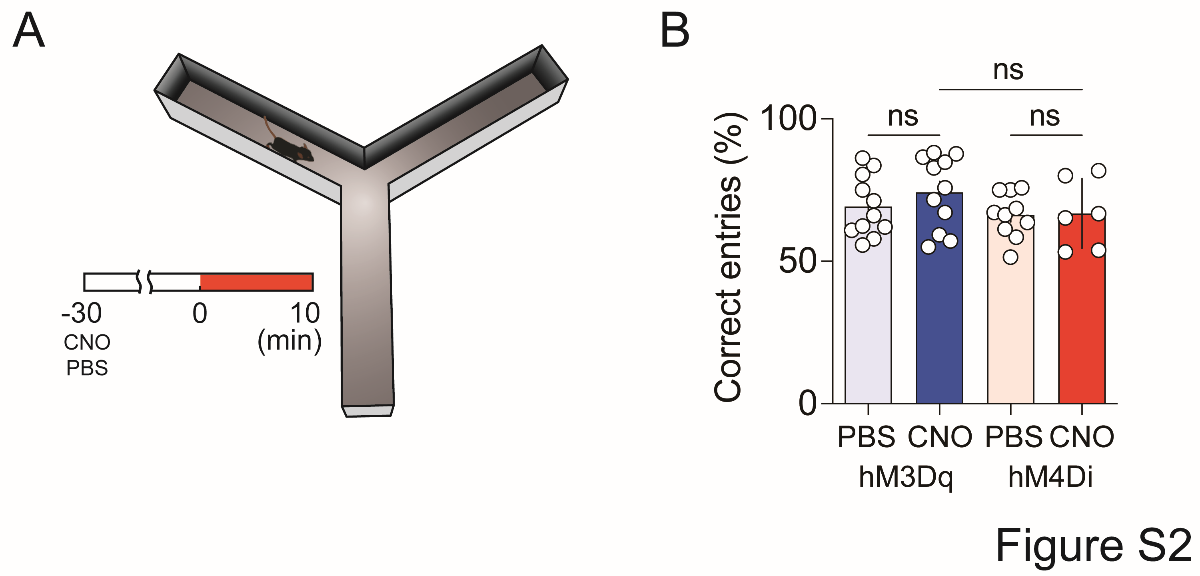


Figure S2. Activity of CaMKII+ neurons in the PH was not involved in spatial working memory. (A) Schematic diagram of the Y-maze test. PBS or CNO was i.p. injected 30 min prior to the Y-maze test. (B) Comparison of the ratio of correct entries in the Y-maze across different mouse groups. hM3Dq-PBS (n = 12), hM3Dq-CNO (n = 11), hM4Di-PBS (n = 10), hM4Di-CNO (n = 6), F_2,35_ = 1.441, P = 0.25, one-way ANOVA followed by Bonferroni’s post hoc test.
